# Exosome in Hepatocellular Carcinoma: an update

**DOI:** 10.7150/jca.54566

**Published:** 2021-03-05

**Authors:** Wei Chen, Yinqi Mao, Chenbin Liu, Han Wu, Shuying Chen

**Affiliations:** 1 Shanghai Jiao Tong University School of Medicine, Shanghai 200025, China.; 2 Department of Laboratory Medicine, Huashan Hospital, Fudan University, Shanghai 200040, China.

**Keywords:** hepatocellular carcinoma, exosome, biological function, biomarker, therapeutic target

## Abstract

Hepatocellular carcinoma (HCC) is a common malignant tumor in the digestive tract with limited therapeutic choices. Intercellular communication among cancer cells and their microenvironment is crucial to disease progression. Exosomes are extracellular vesicles secreted by multiple types of cells into the extracellular space, which contain a variety of active components of secretory cells, including lipids, proteins, RNA and DNA. This vesicle structure involves in the exchange of materials and information between cells and plays an important role in the development of many diseases. Studies have shown that exosomes participate in the communication between HCC cells and non-HCC cells and regulate the occurrence and development of hepatocellular carcinoma. Therefore, exosomes may be specific biomarkers for early diagnosis and metastasis of HCC, which are also potential targets for the treatment of HCC. This review summarizes the characteristic, types and biological functions of exosomes and discusses their research progress and application prospects in the diagnosis and treatment of HCC.

## Introduction

Primary liver cancer (PLC) is one of the most common malignant tumors, and hepatocellular carcinoma is the most common primary liver cancer, accounting for approximately 90% of cases 1. It is the third-largest cause of cancer-related death in the world with high incidence and poor prognosis 2. The development of hepatocellular carcinoma is a complex multi-step process involving continuous inflammatory damage, including liver cell necrosis and regeneration, which is related to fibrous deposition. Although the pathogenesis, molecular characteristics, epidemiology, and other aspects of HCC have made great progress in the past few decades, the molecular classification of hepatocellular carcinoma that has been proposed so far cannot predict the progression and recurrence of the disease 3, and due to the lack of reliable markers, the early diagnosis of HCC is very difficult, and most patients can only be found in the late stage 4. Exosomes are extracellular vesicles with a diameter of 40 nm - 160 nm. They contain many active components of secretory cells, including lipid, protein, RNA, and DNA, and play a key part in the communication between cells 5. By regulating the signal transduction between tumor cells and between tumor cells and non-tumor cells, exosomes indirectly affect the tumor microenvironment and serve as crucial tools in the tumorigenesis 6,7. Multiple studies have shown that exosomes are closely related to the occurrence and development of hepatocellular carcinoma. An in-depth study of exosomes may help decipher the process of tumor formation and metastasis, and provide new methods for early diagnosis and treatment of HCC 8,9.

## Biological characteristics of exosomes

Exosomes are membranous vesicles that are released into the extracellular space by cells after the fusion of intracellular multi vesicles to the cell membrane. They have an extracellular membrane vesicle structure composed of a phospholipid bilayer membrane. Almost all body fluids contain exosomes, including urine, plasma, saliva, bronchial lavage fluid, cerebrospinal fluid, amniotic fluid, ascites, etc. 10. The biological formation mechanism of exosomes has not been fully elucidated, but various cells (normal cells and diseased cells) can secrete exosomes, and compared with normal cells, the release characteristics of tumor cells are more obvious 11.

**Figure 1:**
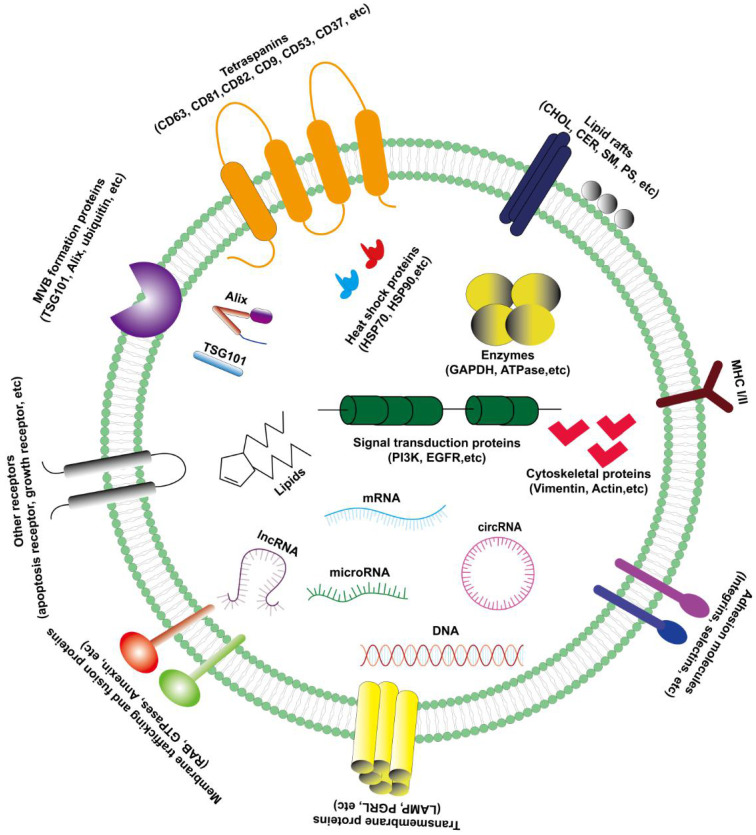
The characteristics and compositions of exosomes. Exosomes are rich in various composition (protein, lipid, RNA and DNA) with extracellular membrane vesicle structure composed of a phospholipid bilayer membrane.

The exosomes are rich in composition, DNA, RNA, lipids, metabolites, and surface protein markers included (Figure 1) 5. The characteristics of exosomes are usually based on their size or the expression of surface markers. There are a variety of characteristic protein components on the outer membrane of exosomes, such as CD63, CD81, CD82, CD9, CD53, CD37, TSG101, Alix and Hsp70, etc. Therefore, western blotting may be used to detect the content of related proteins in the sample to indirectly reflect the presence of exosomes. Besides, exosomes contain various lipid molecules, which not only can participate in various biological processes, but also play an important role in the morphological stability of exosomes in the extracellular fluid: tumor-derived exosomes are protected in the lipid bilayer, thereby protecting their contents (especially nucleic acids) from degradation by extracellular enzymes 12.

Exosomes transmit information through the contents of vesicles and play vital roles in the communication between cells. In the tumor microenvironment, cancer cells produce exosomes in large numbers and transfer from cancer cells to immature cells to participate in cell signal transduction and the process of tumor formation and degradation 13. Studies have shown that exosomes can deliver oncogenes to normal cells under pathological conditions, which may be one of the mechanisms of tumor invasion and metastasis 14. More importantly, the expression of certain proteins and RNA in exosomes is specific to certain tissues and cell types 9. These characteristics of exosomes show that they have great clinical potential as a non-invasive diagnostic method (especially for tumor diagnosis) 15,16.

## Biological components of exosomes

Since exosomes of different cell phenotypes and body fluids contain various biologically active molecules, the types of exosomes are diverse and can be divided into lipids (including cholesterol, sphingomyelin, phosphatidylserine and other lipids) 17, proteins (including oncoproteins, tumor suppressor proteins, and transcription regulators) 18, DNA (including single strand DNA, genomic DNA, and retrotransposon elements) 19,20 and RNA (including messenger RNA (mRNA), micro RNA (miRNA), long non-coding RNA (lncRNA), circular RNA (circRNA) and other non-coding RNA (ncRNA)) (Figure 1) 21. More and more studies have demonstrated that these active components in exosomes play a critical role in a variety of tumors, especially in the progression of hepatocellular carcinoma.

### Exosomal lipids in HCC

Exosomal structure or contents, consisting of lipids, proteins, and nucleic acids, are derived from parent cells. Similar to the cell membrane, lipids are essential components of exosomal membranes 17. Lipids not only have a structural role in exosomal membranes, protecting exosome contents from various stimuli in the circulating fluid, but also are essential players in exosome formation and release to the extracellular environment. Many studies have reported that it is well-known that specific lipids are enriched in exosomes compared to their parent cells. The lipid data for exosomes from PC-3 cells demonstrated a remarkable enrichment from cells to exosomes of cholesterol (CHOL), sphingomyelin (SM), glycosphingolipids (GSL), and phosphatidylserine (PS) 22. Oli-neu cells show less enrichment of SM and a much higher enrichment of ceramide (CER) in exosomes 23. There are also researches focusing on lipids of hepatocellular carcinoma cells. Lipidomic analysis of HepG2/C3a cells showed many similarities with PC-3 cells, contained twice as much free cholesterol and ceramides, three times more phosphatidylserine, over ten times more sphingomyelin, but only one-fifth of the phosphoinositides 24. Haraszti et al. published major differences in the lipid composition for exosomes in Huh7. The free fatty acids most enriched in the exosomes of Huh7 were fully saturated. Cardiolipins, lyso derivatives of phosphatidylserines, phosphatidylglycerols, and phosphatidylinositols also showed enrichment in Huh7 exosomes 25.

In general, both vesicle type and source cell type affect the lipid composition. The diverse content of exosomal lipids makes them an excellent source of noninvasive biomarkers 26. Due to the high stability of the exosomal membrane, it is easier to measure lipids present on the outside of the exosomes than protein and nucleic acid. However, there are few studies on exosomal lipids as biomarkers in hepatocellular carcinoma. With the deepening of exosomal lipid analysis, exosome-derived lipids have considerable potential as biomarkers in the future.

### Exosomal proteins in HCC

The Vesiclepedia database shows that there are as many as 1,800 proteins in exosomes. Among the exosomes derived from HCC cell lines, 213 related proteins were detected by mass spectrometry 27. Besides, exosomes from HepG2 were separated and it was found as many as 1,428 proteins in them by mass spectrometry, which further proved that exosome-derived proteins played an important role in the communication between cancer cells and the microenvironment 28. Dai et al proved by immunohistochemistry, western blotting and quantitative PCR that the reduced level of exosomal CLEC3B in hepatocellular carcinoma promoted metastasis and angiogenesis through AMPK and VEGF signals, which was likely to be a potential therapeutic target for HCC 29. A study on the function of exosomal protein CHI3L1 in hepatocellular carcinoma demonstrated that CHI3L1 activated the TGF-β signaling pathway and promoted tumor progression by affecting gene expression 30. Gai et al confirmed through multiple controlled experiments that the exosomal protein GOLM1 regulated by mTOR/miR-145 regulated the occurrence and metastasis of HCC by activating glycogen synthase kinase-3β/matrix metalloproteinase (GSK-3β/MMPs signal axis), which might be used for targeted treatment 31. Moreover, an increasing number of findings indicate that exosomes selectively enrich tumor-specific proteins. The detection of these proteins may help tumor diagnosis, prognosis, and chemotherapy evaluation. In addition, there are proteins such as Rab27a and SALL-4 that affect the occurrence of HCC by indirectly affecting the secretion of exosomes.

### Exosomal RNA in HCC

Exosome-derived RNAs can be divided into coding RNAs (mRNAs) and non-coding RNAs (ncRNAs). The latter is grouped into miRNAs, lncRNAs and circRNAs according to their length. Non-coding RNA does not have the function of encoding protein but inhibits target RNA through degradation or transformation to regulate post-transcriptional gene expression, relating to development, cell differentiation, proliferation, apoptosis, and inflammation 32. The following will describe in detail the expression of various exosome-derived RNAs in HCC.

#### Messenger RNAs

Messenger RNA is responsible for encoding proteins, and the realization of cell functions are closely related to it. Abd El Gwad and others have verified the excellent ability of exosome-derived RNA as a diagnostic and prognostic marker of HCC through bioinformatics methods. They found that the exosomal lncRNA- RP11-513115.6, miR-1262, and RAB11A mRNA are effective biomarkers for distinguishing HCC patients, and they have higher accuracy when jointly detected, and serum RAB11A mRNA is the most independent predictor 33. Furthermore, a study on the relationship between serum exosomal hnRNPH1 mRNA levels and clinicopathological characteristics of HCC patients showed that high level of serum exosomal hnRNPH1 mRNA in the serum was associated with poor prognosis, which had important predictive value in the prognostic classification of HCC 34.

#### MicroRNAs

MicroRNA is a member of endogenous non-protein-coding RNAs family with a size of approximately 20-22 nucleotides. As a negative regulator of gene expression, it has been found to regulate more than 30% of mRNA and involves in multiple processes such as cell development, differentiation, proliferation, apoptosis and stress 35. All cells in the tumor microenvironment cooperate to play a part in a complex regulatory network, and exosomal miRNAs can regulate the tumor microenvironment and become relevant participants in cell-to-cell communication 36. Lee et al conducted western blot analysis of exosomal markers and found that miR-21 and lncRNA-ATB were independent predictors of HCC, related to the overall survival of patients and disease progression 37. Further research was found that miR-21 inhibited PTEN, leading to the activation of the KT/ERK pathway and causing epithelial-mesenchymal transition (EMT), and miR-10b also had similar effects 38. Moreover, exosomal miR-210 secreted by HCC cells was proved to be transferred to endothelial cells, thereby promoting tumor angiogenesis by targeting SMAD4 and STAT6 39. Similarly, a clinical trials used cell culture, transfection of allogeneic miRNA-224, real-time quantitative PCR, luciferase detection, and other methods verifying that exosomal miR-224 directly targeted the 3'-UTR of GNMT mRNA and influenced the proliferation and invasion of HCC cells 40. In addition to the above, in recent years, more and more exosome-derived miRNAs have been found to be closely related to the pathogenesis and progression of hepatocellular carcinoma. They may not only be used as biomarkers for HCC diagnosis but also provide a new idea for its treatment.

#### Long non-coding RNAs

Similarly, exosomal lncRNAs also make significant contribution to the occurrence, development and transferring of hepatocellular carcinoma. LncRNA is a type of RNA molecules with a transcript length of more than 200 nt. It is generally believed that lncRNAs do not encode proteins, but are involved in protein-coding genes in the form of RNA at multiple levels (epigenetic regulation, transcription regulation, post-transcriptional regulation, etc.) 41. At the earliest, Klingenberg and others applied RNA interference screening methods in HCC cell lines and found that lncRNA CASC9 interacted with HNRNPL to regulate AKT signaling, thereby promoting HCC proliferation 40. Subsequently, the research results on exosomal lncRNAs gradually increased. Using Huh7 cells *in vitro* to simulate the insufficient radiofrequency ablation (RFA), the researcher found that exosomal lncRNA ASMTL-AS1 activated miR-342-3p/NLK/YAP signals, aggravating residual malignant hepatocellular carcinoma when RFA was insufficient, opening up a new way for the treatment of HCC and preventing its recurrence 42. Wang et al. measured the expression of lncRNA H19 in HCC by qRT-PCR and western blot, and found that it could up-regulate LIMK1 and inhibit apoptosis, promote the proliferation, migration, and metastasis of HCC treated with propanol 43. In addition, exosomal lncRNAs are also meaningful for HCC diagnosis. Through a controlled experiment, the levels of exosomal lnc-FAM72D-3 and lnc-EPC1-4 in the serum of HCC patients were found to be abnormally increased, which was differently expressed from the control group 44. These differences make the exosome a potential candidate as reliable biomarkers for hepatocellular carcinoma.

#### Circular RNAs

Circular RNAs are a class of endogenous noncoding RNAs with cell type-specific expression, which function as miRNA sponges to regulate gene expression. They are characterized by their covalently closed loop structures without 5′ caps and 3′ poly tails 45. With the development of deep RNA sequencing (RNA-seq) technologies and novel bioinformatic approaches, abundant and diverse circRNAs have been detected and identified. CircRNAs are abundant and stable in exosomes, and also reported to be associated with the initiation, progression and metastasis of tumors. Wang et al. suggested that circPTGR1 could promote migration and metastasis in HCC through a circPTGR1/miR-449a/MET pathway 46, while miR-449a was described as having roles in cell differentiation and tumor suppression, as well as involvement in the inhibition of tumor growth and metastasis in HCC 47. In addition, circ-0051443 was found to be downregulated in HCC tissues and plasma. Normal cells packaged circ-0051443 into exosomes, secreting them into HCC cells. Exosomal circ-0051443 significantly suppressed the malignant behavior of HCC by reducing the proliferation and migration of cancer cells *in vitro* and *in vivo*. Therefore, exosomal circ-0051443 may be a potential biomarker for the detection of HCC 48. In a recent study, circTMEM45A expression was elevated in HCC and might serve as an oncogene. CircTMEM45A mechanically sponged miR-665 expression, resulting in the upregulation of IGF2 and further promoting the progression of HCC 49. Recent studies have indicated that exosomal circRNAs may have a significant influence on pathophysiologic processes, but only a few of circRNAs have established functional roles or clinical applications. Exosomal circRNAs will be a novel frontier in the research of hepatocellular carcinoma.

### Exosomal DNA in HCC

Currently, although there are few studies on exosomal DNA in HCC, its application in tumor diagnosis is being explored. Yan et al. found that the cfDNA level of HCC patients was significantly higher than that of non-HCC patients 50. In addition, exosome-derived DNA has the advantages of being in the form of high molecular weight and preventing degradation during the cycle 51, which has broad application prospects in serving as a biomarker for screening, diagnosis, treatment and prognosis monitoring of HCC.

## Biological functions of exosomes in HCC

**Figure 2:**
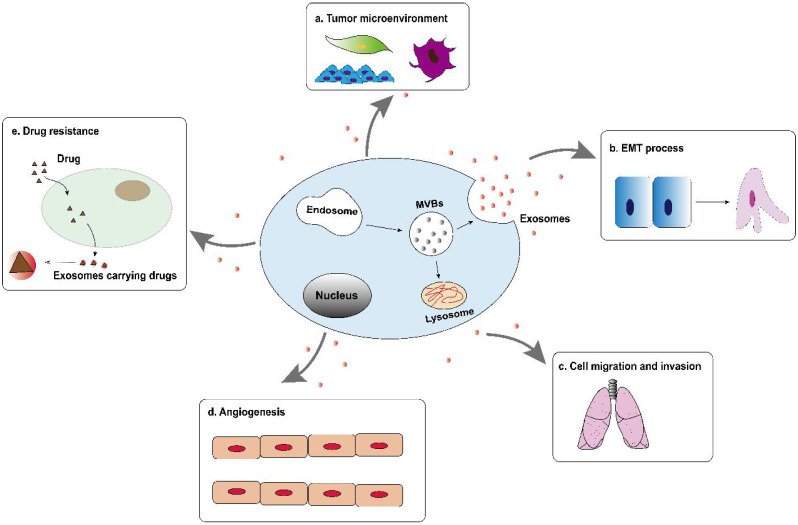
Tumor-derived exosomes are involved in the formation and development of hepatocellular carcinoma, including tumor microenvironment (TME) remodeling, EMT, cell migration and invasion, angiogenesis and drug resistance, etc.

Tumor-derived exosomes are involved in the formation and development of hepatocellular carcinoma, including tumor microenvironment (TME) remodeling, EMT, cell migration and invasion, angiogenesis and drug resistance, etc. (Figure 2). Exosomes activate or inhibit various signal transduction pathways in recipient cells by transmitting biologically active cargo, thereby regulating a variety of malignant behaviors of hepatocellular carcinoma cells. Therefore, understanding the roles of exosomes in the biology of hepatocellular carcinoma will help develop new anti-tumor strategies.

### Exosomes and tumor microenvironment

Tumor microenvironment is the cellular environment of tumor in human body. In addition to tumor cells, TME also includes a variety of stromal cells (endothelial cells, immune cells, adipocytes, cancer-related fibroblasts and mesenchymal cells, etc.) and extracellular matrix (cytokines, chemokines and growth factors, etc.) 52. Tumor growth and metastasis are highly dependent on the interaction between tumor and its related microenvironment. For tumor growth, it is necessary not only to communicate between tumor cells, but also to crosstalk between cancer cells and stromal cells in TME. Exosomes are important components of tumor microenvironment mediating cell-cell interaction. At present, there are three main forms of communication between exosomes and cells: exosomes act as signal complexes to stimulate target cells, exosomes transfer receptors between cells and exosomes deliver functional proteins, nucleic acids or lipids to receptor cells 53. In HCC, exosomes significantly affect the progression of the disease by regulating the two-way communication between tumor cells and their surrounding microenvironment. A study on hepatoma cells and hepatic stellate cells in tumor microenvironment showed that hepatocellular carcinoma cells could directly secrete the exosomal miR-21 by targeting PTEN, leading to the activation of PDK1/Akt in HSC, thus further transforming the quiescent hepatic stellate cells into activated cancer associated fibroblasts (CAFs). Activated CAFs promoted the progression of tumor by secreting angiogenic cytokines (including VEGF, MMP2, MMP9, bFGF and TGF-β) 54. Another study found that the exosomes derived from HepG2 could be actively internalized by adipocytes differentiated from mesenchymal stem cells (MSCs), activated NF-κB signaling pathway and various kinases in adipocytes, and caused significant transcriptome changes (especially the inflammatory phenotype in adipocytes). After treatment with macrophages, the proliferation of tumor cells was further enhanced. The adipocytes treated with tumor-derived exosomes enhanced angiogenesis and recruited more macrophages, further promoting tumor growth 28. More and more studies have provided critical roles of exosomes in regulating the dynamic crosstalk between different cell populations in TME, providing an important theoretical basis and an anti-tumor strategy target for further understanding the mechanism of the occurrence and development of hepatocellular carcinoma.

### Exosomes and epithelial mesenchymal transition

Epithelial mesenchymal transition is a process where epithelial cells are stimulated by extracellular factors, lose their adhesion properties and then transform into mesenchymal cells. It gives cells the ability to metastasize and invade. It is considered an important step in the formation and progression of tumor 55. More and more evidences indicate that exosomes may carry a variety of EMT precursors including EMT inducer molecules, which become important mediators of EMT process by activating related pathways. For example, after treatment with exosomes derived from HCC cells, the expression of EMT promoters (ZEB1, ZEB2, and Slug) and interstitial related markers (N-Cadherin, α-SMA and Vimentin) in liver cancer cells increased , While the expression of the promoter (OVOL1) driven by epithelial markers (E-cadherin) and mesenchymal-epithelial transition (MET) was reduced 56. In addition, when recipient HCC cells were co-cultured with exosomes derived from MHCC97L or MHCC97H cells, the expression of E-cadherin decreased, while the expression of the mesenchymal phenotype protein (Vimentin) increased, indicating that the recipient HCC cells experienced EMT 57. The study by Yang et al. found that tumor-derived exosomal miR-92a-3p increased the expression of mesenchymal biomarkers (N-cadherin, β-catenin and Snail) by regulating the PTEN/AKT pathway, and at the same time decreased the protein level of E-cadherin, which promotes the EMT process and plays a key role in the metastasis of HCC 58. Therefore, exosomes derived from HCC cells or stromal cells can drive the phenotypic changes of recipient cells through autocrine or paracrine mode, thereby promoting the progression and metastasis of HCC.

### Exosomes and cell migration and invasion

Tumor cells from the primary tumor to surrounding tissues is an important link to initiate cancer metastasis, and metastasis is the main cause of cancer-related death 59. The process of cell migration and invasion involves many complex processes. As an important communication tool in the tumor microenvironment, exosomes have made great contributions in all stages of liver cancer cell migration and invasion. Exosomal-derived LOXL4 activated the FAK/Src pathway through a hydrogen peroxide-mediated mechanism to promote cell matrix adhesion, cell migration and invasion that depend on its catalytic activity to promote the distant metastasis of hepatocellular carcinoma 60. In addition, compared with low-metastatic HCC cells, exosomal circRNA-100,338 is over-expressed in high-metastatic HCC cells, and high-expressed exosomal circRNA-100,338 significantly promote the migration and invasion of cells 61. Another study found that exosomal SENP3-EIF4A1 can metastasize from normal cells to hepatocellular carcinoma cells. As an important communication tool between cells, exosomal SENP3-EIF4A compete with miR-9-5p to regulate the expression of downstream target gene ZFP36, inhibit the migration and invasion of hepatocellular carcinoma cells, and further block the progression of HCC 62. Although these data provide substantial new evidence that exosomes are involved in liver cancer metastasis, their upstream regulators or downstream signaling cascades in the metastasis of the tumor have not yet been fully elucidated. Therefore, the identification of exosomes that are critical to the progression of hepatocellular carcinoma can not only better understand how exosomes control cancer invasion and metastasis, but also may find preferred markers that may be used for liver cancer diagnosis and prediction.

### Exosomes and angiogenesis

Tumor metastasis not only requires the participation of tumor cell migration and invasion, but also involves tumor cells cross the endothelial barrier. Since Judah Folkman put forward the hypothesis that tumor growth requires angiogenesis in 1971 63. More and more studies have found that on the one hand, tumor-related neovascular system provides relevant oxygen and nutrients for growing tumors, on the other hand, abnormal vascular structure in tumor lesions leads to increased vascular permeability, conducive to tumor growth and metastasis 64. Hepatocellular carcinoma is a typical hypervascular tumor. Tumor-derived exosomes can transfer biologically active substances to vascular endothelial cells, mediate the crosstalk between cancer cells and endothelial cells, and induce angiogenesis. Recently, it has been discovered that exosomes participate in the regulation of various stages of liver cancer angiogenesis. Exosomes derived from liver cancer cells carry angiopoietin 2 (ANGPT2), which is transported to vascular endothelial cells through exosomal endocytosis, resulting in a significant increase in angiogenesis. CRISPR-Cas system knocking out ANGPT2 can significantly inhibit angiogenesis induced by exosomal ANGPT2 secreted by HCC cells 65. Another study found that under hypoxic conditions, exosomal miR-155 derived from HCC cells can induce HUVEC angiogenesis, and affect the angiogenic activity and recurrence of hepatocellular carcinoma. Although the study has not further determined the specific signaling pathway downstream of miR-155, clinical statistics show that the high expression of exosomal miR-155 in the preoperative plasma of patients with HCC is significantly related to the early recurrence and metastasis of patients, and the results of immunohistochemistry further confirmed that the high expression of exosomal miR-155 was significantly correlated with the expression of VEGF, HIF-1α, and MVD 66. Therefore, in the angiogenesis of HCC, treatment intervention based on exosomes will help the body maintain the dynamic balance of anti-vascular and pro-vascular.

### Exosomes and drug resistance

Drug resistance is an important factor that affects the correct treatment and prognosis of patients, and it is also an urgent issue for clinical diagnosis and treatment. As a new type of molecular marker, exosomes have been reported to be closely related to drug resistance in tumor. Generally, the main function of exosomes in normal cells is to remove unfavorable factors for the cells. In tumor cells, exosomes mediate multiple drug resistance mechanisms to confer cytochemical resistance, including by inducing EMT to participate in drug resistance, by promoting anti-apoptotic pathways or changing signal transduction pathways to obtain chemotherapy resistance, by directly or indirectly regulating the drug efflux pump to influence chemical drug resistance and change tumor chemical resistance through immune cells, cancer stem cells and other stromal cells 67. Hepatocellular carcinoma seriously affects human life and health, and chemotherapy has become the standard method of non-surgical treatment for hepatocellular carcinoma. However, the emergence of drug resistance in HCC cells often hinders the therapeutic effects of chemical drugs. Studies on HCC cells have shown that exosomal miR-199a-3p reversed tumor chemoresistance and reduced the resistance of HCC to cisplatin 68. The exosomes of mesenchymal stem cells derived from adipose tissue effectively mediated the delivery of miR-199a to HCC cells, while AMSC-Exo-199a targeted mTOR and subsequently inhibited the mTOR pathway, making HCC cells sensitive to adriamycin. Intravenous AMSC-Exo-199a might be distributed to tumor tissues and significantly improved the effect of doxorubicin on hepatocellular carcinoma *in vivo* 69. Moreover, when HCC cells were exposed to multiple anticancer drugs, lincRNA-VLDLR could be up-regulated in both hepatocellular carcinoma cells and extracellular vesicles. In extracellular vesicles, linc-VLDLR increased the ATP-binding cassette G subfamily 2 member (ABC-G2) expression and then reduced cell death induced by sorafenib and other chemotherapy drugs 70. As a research hotspot, the discovery of exosomes provides new insights into the potential mechanism of tumor cell resistance to chemotherapy drugs. These findings enrich the research of exosomes in regulating the sensitivity of hepatocellular carcinoma chemotherapeutics, and also provide the possibility for the future targeted treatment of hepatocellular carcinoma through exosomes as drug carriers.

## Clinical applications of exosomes in HCC

Exosomes change the development process of HCC by providing important biologically active molecules in different stages of HCC progression, which also indicates that exosomes have the potential as diagnostic biomarkers and therapeutic targets. Table 1 has listed the expression of different exosomes in hepatocellular carcinoma. In addition, since the membrane composition of exosomes is similar to that of cells in the body and does not cause an immune response, it can be applied to the delivery system of anticancer drugs 71.

### Exosomes are potential biomarkers of HCC

Due to hepatocellular carcinoma with no specific manifestations in the early stage generally, patients often miss the optimal treatment period. Therefore, early diagnosis is the most important prerequisite for successful treatment of hepatocellular carcinoma. Current guidelines recommend serum alpha-fetoprotein (AFP) and ultrasonography (US) at a regular basis for the early detection of HCC in the at-risk population 72. The former has low sensitivity and specificity for HCC screening 73, while the latter has a limited application range and cannot obtain the full and dynamic information of tumor tissues. As a classic biomarker of hepatocellular carcinoma, AFP remains the most widely used and accepted serum marker since its discovery over 60 years ago 74. However, elevated AFP is not unique to hepatocellular carcinoma. AFP can also appear in the serum of patients with gastric cancer, pancreatic cancer, lung cancer and other tumors. Besides, AFP in the serum of patients with chronic hepatitis B or liver cirrhosis may also increase. The emergence of exosomes provides new ideas for the early diagnosis of HCC. There are many types of exosomes from different tumor sources, and their content is significantly different compared with healthy people 75,76. More importantly, compared with the complex environment of tissues and cells, the environment of exosomes is relatively stable, so they can be used for the diagnosis of HCC progression and metastasis 50. A classic study has shown that exosomes are one of the extension factors of tumors, and the expression of tumor-derived exosomes can be used to predict tumor metastasis to specific organs 77. Arbelaiz and others analyzed the expression of exosomal proteins in the blood of HCC patients and healthy people. They found that G3BP and PIGR were significantly increased in the exosomes of HCC patients 74. In addition to protein, recent studies have also shown that the levels of exosomal miR-21, miR-224, miR-210 and miR-93 in the serum of HCC were significantly higher than those of healthy subjects 38-40,78, while the levels of miR-9-3p and miR-638 were significantly lower 79,80. However, the level of exosomes alone is often unable to accurately predict the development of HCC, and the analysis of multiple factors including AFP may better diagnose HCC. In addition, researches have been reported that exosomes can be used for prognostic biomarker for HCC. The evaluation in the prognostic value of serum exosomal microRNAs revealed that patients with overexpressed serum exo-miR-215-5p had significantly poor DFS compared to patients with low expression of serum exo-miR-215-5p 81. In the experiments by Yugawa 82, exosomes were characterized using western blotting, TEM, and NanoSight analysis. They found that overexpression of miR-150-3p significantly inhibited migration and invasiveness of both Huh7 and Hep3B cells, while patients with low miR-150-3p expression had significantly worse prognosis. MiR-150-3p was a significantly prognostic factor in HCC patients. In brief, the exosome-derived biomarkers may be applied on the disease diagnosis and prognosis judgment.

### Exosomes are therapeutic targets of HCC

The application of exosomes in the treatment of HCC mainly includes two points. On the one hand, the tumor suppressor contained in the exosomes can directly exhibit tumor suppression effects. For example, studies have shown that exosomal miR-122 produced by hepatocellular carcinoma can inhibit EMT, increase drug sensitivity, and inhibit angiogenesis by targeting LMNB2 83,84. On the other hand, because exosomes are lipid-like and easy to metastasize, their stable membrane structure is certainly an advantage for the stability of such vehicles following intravenous injection. They are likely to be effective drug delivery tools. Existing studies have confirmed that exosomes-based drug delivery methods can effectively inhibit tumor growth 85. However, in the current various clinical cancer treatments including hepatocellular carcinoma, there are still many difficulties to be overcome in using exosomes as carriers. It is believed that with the continuous improvement of exosomes extraction, identification, and purification technology in the future, exosomes will be widely used in the treatment of HCC.

In addition, immunotherapy is becoming a new treatment method for advanced hepatocellular carcinoma worldwide. Hepatocarcinoma cell-derived exosomal circUHRF1 are delivered to NK cells to up-regulate TIM-3 expression by sponging miR-449c-5p, thereby inducing NK cell dysfunction and promoting immune suppression. And the overexpression of exosomal circUHRF1 further prevent the anti-tumor effect of PD1 treatment (Opdivo). Therefore, targeting exosomal circUHRF1 may be a promising and effective method to restore the sensitivity of HCC to anti-PD1 therapy 86. Another study on dendritic cells and immunotherapy found that dendritic cell exosomes rich in AFP may trigger an effective antigen-specific anti-tumor immune response and reshape the tumor microenvironment of HCC mice, providing a cell-free vaccine option HCC immunotherapy 87. In short, exosomes show great potential in drug resistance and immunotherapy, and can provide potential treatment strategies for the clinical application of hepatocellular carcinoma.

## Conclusion

Exosomes participate in the communication between HCC cells and non-HCC cells. By regulating key targets, they can affect the occurrence and development of hepatocellular carcinoma. The proteins, nucleic acids, and other substances contained in exosomes exist in a relatively independent special environment and have higher stability and abundance than tumor markers existing in tissues or body fluids. Therefore, exosomes have unique advantages in the early diagnosis of HCC. They can also be used as therapeutic targets for the treatment of HCC, and they even serve as carriers to achieve targeted drug delivery. However, due to problems in the identification, isolation, and purification of exosomes, unclear molecular mechanisms, and immature synthetic exosomes technology, research on the formation mechanism of HCC by exosomes is still not deep enough, and the diagnosis and treatment of exosomes related to HCC are still in the preclinical experimental stage. With more research conducted on exosomes, it is believed that exosomes could bring breakthroughs and transformative changes in the diagnosis and treatment of HCC soon.

## Figures and Tables

**Table 1:** The expression of different exosomes in hepatocellular carcinoma

Contents of Exosomes	Molecules	Actions	References
Protein	CLEC3B	Prevent metastasis and angiogenesis	29
	CHI3L1	Activate TGF-β signaling pathway	30
	GOLM1	Accelerate cell proliferation and migration	31
Messenger RNA	RAB11A	Poor prognosis	18
	hnRNPH1	Portal vein tumor emboli and lymph node metastasis	34
MicroRNA	miR-21	Proliferation and Metastasis, EMT	37,38
	miR-210	Angiogenesis	39
	miR-224	Cell proliferation	40
	miR-1262	Poor prognosis	18
	miR-93	Poor prognosis	78
	miR-9-3p	Suppresse HBGF-5 expression and induce cell apoptosis	79
	miR-122	Inhibit hepatocellular carcinoma cell progression by targeting LMNB2	84
	miR449a	cell differentiation and tumor suppression	47
	miR-10b-5p	suppressing tumor suppressor genes	81
	miR-215-5p	vascular invasion and poor prognosis	81
	miR-150-3p	inhibits HCC migration and invasiveness	88
Long noncoding RNA	lncRNA CASC9	Cell proliferation	89
	lncRNA ASMTL-AS1	Activate YAP signalling and Exacerbate tumor progression	42
	lncRNA H19	Upregulate LIMK1 and promote the proliferation, migration and invasion	43
	lnc-EPC1-4	Inhibite cell proliferation	44
	lnc-FAM72D-3	Induce cell apoptosis	44
Circular RNA	circPTGR1	promote migration and metastasis	46
	circ0051443	reduce the proliferation and migration of cancer cells	48
	circTMEM45A	upregulate the IGF2 and promote HCC progression	49
DNA	cfDNA	improve the diagnostic performance of HCC	50

## Abbreviations

HCC: hepatocellular carcinoma
PLC: primary liver cancer
mRNA: messenger RNA
miRNA: micro RNA
lncRNA: long non-coding RNA
circRNA: circular RNA
ncRNA: non-coding RNA
CHOL: cholesterol
SM: sphingomyelin
GSL: glycosphingolipids
PS: phosphatidylserine
CER: ceramide
RFA: radiofrequency ablation
EMT: epithelial-mesenchymal transition
TME: tumor microenvironment
CAFs: cancer associated fibroblasts
MSCs: mesenchymal stem cells
MET: mesenchymal-epithelial transition
ANGPT2: angiopoietin 2
AFP: alpha-fetoprotein
US: ultrasonography (US)
